# Pregnancy and Childbirth in Women With Meningioma

**DOI:** 10.7759/cureus.27528

**Published:** 2022-07-31

**Authors:** David R Hallan, Debarati Bhanja, Bao Y Sciscent, Casey Ryan, Michael J Gigliotti, Lekhaj C Daggubati, Catherine Caldwell, Elias Rizk

**Affiliations:** 1 Neurosurgery, Penn State Health Milton S. Hershey Medical Center, Hershey, USA

**Keywords:** obstetrics, childbirth, pregnancy, outcomes, meningioma, neurosurgery

## Abstract

Background

Ten percent of women of childbearing age have histologically confirmed meningioma. To date, little is known regarding pregnancy-related outcomes for women with meningioma.

Methods

We used a de-identified database network (TriNetX's Research Network, https://trinetx.com/) to gather information on pregnant patients with meningioma (cohort 1) versus pregnant patients without meningioma (cohort 2). The primary outcome of interest included the impact of meningioma on mortality at one year. Secondary endpoints included ectopic or molar pregnancy, cesarean section, abortion, preterm labor, depression, pre-eclampsia/eclampsia, and craniotomy. Odds ratios (OR) with 95% confidence intervals (CI) were used to measure levels of association between each cohort and the outcomes of interest.

Results

A total of 1,739 patients were identified in each cohort following propensity-score matching. Mortality was seen in 23 patients (1.32%) in cohort 1 versus 26 patients (1.41%) in cohort 2 (OR 0.88, 95% CI {0.50, 1.55}, p=0.66). Ectopic/ molar pregnancy was seen in 31 (1.78%) versus 42 (2.42%) patients in cohorts 1 and 2, respectively (OR 0.73, 95% CI {0.046,1.17}, p=0.19). Cesarean section was seen in 126 (7.25%) versus 164 (9.43%) patients, respectively (OR 0.75, 95% CI {0.59,0.97}, p=0.020). Abortion was seen in 128 (7.36%) versus 183 (10.52%) patients, respectively (OR 0.68, 95% CI {0.53,0.86}, p=0.0011). Preterm labor was seen in 75 (4.31%) versus 119 (6.84%) patients, respectively (OR 0.61, 95% CI {0.46,0.83}, p=0.0012). Depression was seen in 258 (14.84%) versus 270 (15.53%) patients, respectively (OR 0.95, 95% CI {0.79,1.14}, p=0.57). Pre-eclampsia/eclampsia was seen in 3.11% versus 5.52% patients, respectively (OR 0.55, 95% CI {0.39,0.77}, p=0.0005). Craniotomy was seen in 74 (4.26%) versus 0 (0%) patients in cohort 1 and cohort 2, respectively.

Conclusion

Patients with meningioma were not at higher risk for pregnancy complications, including ectopic/molar pregnancy, cesarean section, abortion, preterm labor, pre-eclampsia/eclampsia, and mortality, compared to their non-meningioma counterparts. Still, coordinated care by neurosurgical and obstetrical providers may benefit women with meningiomas who are planning for pregnancy or are currently pregnant.

## Introduction

Meningioma is the most common tumor in the brain [1]. Meningiomas are typically diagnosed in females between the ages of 40-60, but nearly 30% of women with meningiomas are of childbearing age [1,2]. To date, there is limited literature on pregnancy-related outcomes for women with meningioma, and of the literature available much is associated with a mixed clinical course. The objective of this study is to compare pregnancy outcomes, gestational conditions, and mortality between women with and without meningioma.

## Materials and methods

Database query

We retrospectively queried International Classification of Disease (ICD-10) codes and procedural codes via Common Procedural Terminology (CPT) codes on TriNetX (https://trinetx.com/), a de-identified database network, to evaluate all patients with a concomitant diagnosis of meningioma and pregnancy (cohort 1) versus pregnant patients without a concomitant meningioma diagnosis (cohort 2). Data came from 58 health care organizations (HCOs) spanning six countries. Data includes demographics, diagnoses, medications, laboratory values, genomics, and procedures. The identity of the HCOs and patients is not disclosed to comply with ethical guidelines against data re-identification. Because of the database's federated nature, an Institutional Review Board (IRB) waiver has been granted. The data is updated daily. Previous literature informed our use of this database and its validity, and the network's exact details have been previously described [3-6]. The diagnosis was based on ICD-10 and CPT codes. The index date was set at the date of pregnancy.

Propensity-score matching

To adjust for hypothesized confounders on the relationship between meningioma and pregnancy outcomes of interest, medical information including age at pregnancy, as well as sex, race, and comorbidities of hypertension, obesity, hypothyroidism, diabetes, asthma, migraine, epilepsy, prior pregnancy with abortive outcome, nicotine dependence, coagulation defects, edema, chronic obstructive pulmonary disease, history of spontaneous abortion, thrombocytopenia, history of prior ectopic pregnancy, antiphospholipid syndrome, prior missed abortion, systemic lupus erythematous, prior primary inadequate contractions, and prior induced abortions were recorded up until the date of index.

Outcome measures

Our primary endpoint was mortality at one year from pregnancy. Secondary endpoints included ectopic or molar pregnancy, cesarean section, abortion, preterm labor, depression, pre-eclampsia/eclampsia, and craniotomy. Analysis was performed using unmatched and propensity score-matched cohorts, with the greedy-nearest neighbor algorithm with a caliper of 0.1 pooled standard deviations. Chi-square analysis was performed on categorical variables.

## Results

After propensity score matching, 1,739 patients were identified in each cohort (Table 1). Cohort 1 represents patients with meningioma, and cohort 2 represents patients without meningioma. Age at pregnancy was 42.2±17.0 years and 42±17.9 years for cohorts 1 and 2, respectively. 61.19% versus 62.80% of patients were white, 18.63% versus 18.86% were black or African American, and 17.42% versus 15.58% were of unknown race. Baseline demographics and characteristics are shown in Table 1. Variables with less than 10 patients or without significant differences prior to matching were not included.

**Table 1:** Baseline demographics and characteristics for patients with meningioma (cohort 1) and without meningioma (cohort 2) before and after propensity score matching.

Variable	Unmatched		Matched	
	Cohort 1: Meningioma, N (%)	Cohort 2: No meningioma, N (%)	Cohort 1: Meningioma, N (%)	Cohort 2: No meningioma, N (%)
Patients (n)	1,762	26,168	1,739	1,739
Age at Index	42.7 ± 17.5	32.1 ± 12.5	42.2 ± 17	42 ± 17.9
Race				
White	1,074 (60.95)	13,029 (49.79)	1,064 (61.19)	1,092 (62.80)
Black	325 (18.45)	9,853 (37.65)	324 (18.63)	328 (18.86)
Asian	3141 (7.82)	2,551 (9.75)	303 (17.42)	271 (15.58)
Unknown	39 (2.21)	588 (2.25)	38 (2.19)	33 (1.90)
Comorbid Conditions				
Hypertension	479 (27.19)	5,287 (20.20)	464 (26.68)	439 (25.24)
Obesity	286 (16.23)	8,019 (30.64)	286 (16.45)	240 (13.80)
Diabetes Mellitus	199 (11.29)	3,066 (11.72)	194 (11.16)	190 (10.93)
Supervision of high-risk pregnancy	300 (17.03)	7,934 (30.32)	300 (17.25)	288 (13.80)
Migraine	192 (10.90)	2,952 (11.28)	192 (11.04)	166 (9.55)
Asthma	194 (11.01)	3,964 (15.15)	194 (11.16)	173 (9.95)
Epilepsy or recurrent seizures	143 (8.12)	522 (2.00)	129 (7.42)	115 (6.61)
Coagulation Disorders	121 (6.87)	1,118 (4.27)	114 (6.56)	114 (6.56)
Chronic Obstructive Pulmonary Disease	82 (4.65)	661 (2.53)	79 (4.54)	60 (3.45)
Systemic Lupus Erythematous	13 (0.74)	337 (1.29)	12 (0.69)	15 (0.86)

For pregnancy outcomes (Table 2), ectopic/ molar pregnancy was seen in 31 (1.78%) versus 42 (2.42%) patients in cohorts 1 and 2, respectively (OR 0.73, 95% CI {0.046, 1.17}, p=0.19). Cesarean section was seen in 126 (7.25%) versus 164 (9.43%) patients, respectively (OR 0.75, 95% CI {0.59, 0.97}, p=0.020). Abortion was seen in 128 (7.36%) versus 183 (10.52%) patients, respectively (OR 0.68, 95% CI {0.53, 0.86}, p=0.0011). Preterm labor was seen in 75 (4.31%) versus 119 (6.84%) patients, respectively (OR 0.61, 95% CI {0.46, 0.83}, p=0.0012). Depression was seen in 258 (14.84%) versus 270 (15.53%) patients, respectively (OR 0.95, 95% CI {0.79, 1.14}, p=0.57). For gestational conditions, pre-eclampsia/eclampsia was seen in 3.11% versus 5.52% patients, respectively (OR 0.55, 95% CI {0.39, 0.77}, p=0.0005). For meningioma-related outcomes, craniotomy was seen in 74 (4.26%) of patients in cohort 1 versus 0 in cohort 2. Finally, mortality was seen in 23 (1.32%) versus 26 (1.41%) patients, respectively. Table 2 shows outcomes after propensity score matching.

**Table 2:** Pregnancy, gestational, and survival outcomes after propensity score matching between patients with meningioma (cohort 1) and without meningioma (cohort 2). CI: Confidence Interval

Outcome	Cohort 1: Meningioma, n (%)	Cohort 2: No Meningioma, n (%)	Odds ratio (95% CI)	P-value
Ectopic or molar pregnancy	31 (1.78)	42 (2.42)	0.733 (0.459, 1.172)	0.1932
Cesarean section	126 (7.25)	164 (9.43)	0.75 (0.589, 0.956)	0.0198
Abortion	128 (7.36)	183 (10.52)	0.676 (0.533, 0.856)	0.0011
Preterm labor	75 (4.31)	119 (6.84)	0.614 (0.456, 0.826)	0.0012
Depression	258 (14.84)	270 (15.53)	0.948 (0.788, 1.141)	0.5707
Pre-eclampsia/Eclampsia	54 (3.11)	96 (5.52)	0.548 (0.39, 0.771)	0.0005
Craniotomy	74 (4.26)	0 (0)	-	< 0.0001
Mortality	23 (1.323)	26 (1.495)	0.883 (0.502, 1.554)	0.666

## Discussion

Pregnancy has been shown to increase meningioma growth and symptomatology. This is likely due to a hyperhormonal state (increases in progesterone, human placental lactogen, and prolactin), as well as water retention and engorgement of vessels [1,7-9]. Histopathology of these tumors has shown the expression of estrogen (ER+) and progesterone (PR+) receptors [10,11]. Meningiomas likewise have been shown to grow faster during the luteal phase of menstruation [8]. It was long thought that pregnancy also increased the incidence of meningiomas, although this has shown to not be the case, with the incidence of meningioma either similar or decreased [7,12,13]. A 2021 study by Pettersson‑Segerlind et al. utilized the Swedish National Population Registry and compared the risk of developing a meningioma both during and after pregnancy [13]. They found an increased incidence of meningiomas in nulliparous women (standardized incident ratio = 1.73, 95% CI {1.52-1.95}). Also, the number of cases of meningioma detected during pregnancy was lower than expected (standardized incidence ratio=0.40, 95% CI {0.20-0.72}), and there was no increased risk of meningioma formation after one-year post-partum (standardized incidence ratio = 1.04; 95% CI {0.74-1.41}) [13].

The American College of Obstetricians and Gynecologists (ACOG) recommends that a pregnant woman should not be denied or delayed necessary surgery regardless of trimester. However, unfortunately, no guidelines exist on the best management of meningioma in pregnancy, which can make prenatal and peripartum care more difficult [1,7,12]. The diagnosis and severity of meningioma during pregnancy can be challenging. Symptoms of high intracranial pressure, such as vomiting, may be misconstrued as hyperemesis gravidarium and seizure activity, which can occur from eclampsia or from a tumor [7,12]. Furthermore, a head computed tomography (CT) scan is unlikely to be pursued during pregnancy due to the effects of radiation. However, what is known about meningiomas and pregnancy is that brain surgery during pregnancy is very risky, both for the mother and the child [9,14]. One study showed an increased mortality odds ratio as high as 14.7 for surgery during pregnancy compared to after pregnancy [14]. The risks and benefits of surgery must be weighed carefully taking into consideration the patient’s presentation and tumor growth. In addition to the obvious need for multidisciplinary decision-making in these situations, prior literature has suggested the need for biophysical profiles and cardiotocography, that gestation should be prolonged for as long as possible, that elective cesarean section is preferred in order to mitigate the risk of increased intracranial pressure during delivery, and that if patients are doing well clinically then tumor resection should be delayed until after childbirth [1,7,8]. It should also be noted that mannitol should only be used when its benefit significantly outweighs risk since it can cross the maternal-fetal barrier, significantly decrease uteroplacental blood flow, and because anesthetic agents may cause unwanted effects on the fetus especially if they are less than 12 weeks and organogenesis is incomplete [1,9].

In 2018, Laviv et al. published a literature review looking at the clinical outcome of 104 cases of meningioma in pregnancy and divided patients into 2 cohorts: those who had a craniotomy during pregnancy or at delivery and those who underwent surgery after delivery [14]. They found an increased mortality rate of both the mom and the fetus in those who underwent craniotomy during pregnancy, with an odds ratio of 14.7, although that group had far more emergent craniotomies (40% vs 19.6%) and emergent cesarean sections (47% vs 17.8%) than the group who had surgery after delivery. In this study, the rate of premature delivery was high in both groups at around 70% [14]. This study did not have nearly as high a rate of premature delivery nor the number of craniotomies performed; the high rate of premature delivery and surgeries performed may reflect publication bias.

In 2012, Lusis et al. published a case series of 17 patients with meningioma resected during pregnancy [15]. They found that 16 of those patients survived, and the only death was attributed to prior complications [15]. Our study found similar mortality rates for both the meningioma-in-pregnancy group and pregnancy alone groups.

While it has been shown that meningiomas increase in size and patients become more symptomatic during pregnancy, this study shows that the rates of ectopic/molar pregnancy, cesarean section, abortion, preterm labor, pre-eclampsia/eclampsia, and mortality do not dramatically differ from non-meningioma pregnant patients. Future analyses should explore relationships between these outcomes and gravida number for pregnant women with meningioma.

Our analysis was limited due to its retrospective nature. In addition, we were unable to collect patient-level data on specific outcomes. We were unable to report on radiology information regarding the anatomic location of the tumor and on histological grading of the meningiomas nor the methodology that was utilized to identify histologic subtype. The data collected was for billing purposes, not for clinical use, and thus much clinical information is missing. In addition, some misidentification is inevitable in database studies.

## Conclusions

This analysis found that pregnant women with meningioma were not at higher risk for pregnancy complications compared to their non-meningioma counterparts. Rates of ectopic/molar pregnancy, cesarean section, abortion, preterm labor, pre-eclampsia/eclampsia, and mortality were not dramatically different between the two groups. Nonetheless, women with meningioma who are currently pregnant or planning for pregnancy may benefit from coordinated care by neurosurgical and obstetrical providers.
